# Effects of GHRP-2 and Cysteamine Administration on Growth Performance, Somatotropic Axis Hormone and Muscle Protein Deposition in Yaks (*Bos grunniens*) with Growth Retardation

**DOI:** 10.1371/journal.pone.0149461

**Published:** 2016-02-19

**Authors:** Rui Hu, Zhisheng Wang, Quanhui Peng, Huawei Zou, Hongze Wang, Xiaoqiang Yu, Xiaoping Jing, Yixin Wang, Binghai Cao, Shanke Bao, Wenhua Zhang, Suonan Zhao, Hanzhong Ji, Xiangying Kong, Quanxi Niu

**Affiliations:** 1 Institute of Animal Nutrition, Sichuan Agricultural University, Key Laboratory of Low Carbon Culture and Safety Production in Cattle in Sichuan, Chengdu, Sichuan, P.R. China; 2 College of Animal Science and Technology, State Key Laboratory of Animal Nutrition, China Agricultural University, Beijing, P.R. China; 3 Animal Husbandry and Veterinary Institute, Haibei, Qinghai, P.R. China; 4 Ningxia Xiahua Muslim Food Co. Ltd., Zhongwei, Ningxia, P.R. China; 5 Institute of Animal Genetics and Breeding, College of Animal Science and Technology, Sichuan Agricultural University, Chengdu, Sichuan, P.R. China; University of Macau, MACAO

## Abstract

The objective of this study was to investigate the effects of growth hormone-releasing peptide-2 (GHRP-2) and cysteamine (CS) administration on growth performance in yaks with growth retardation and try to elucidate its regulatory mechanisms. Trial 1, thirty-six 1-year-old Qinghai high plateau yaks (body weight 38–83.2 kg) were randomly chosen for body weight and jugular blood samples collection. The relationship between body weight and serum GHRH (*P* < 0.05, R = 0.45), GH (*P* < 0.05, R = 0.47), IGF-1 (*P* < 0.05, R = 0.62) was significantly correlated in yaks colonies with lighter body weights. Trial 2, fifteen 1-year-old Qinghai high plateau yaks with growth retardation (average body weight 54.8 ± 8.24 kg) were randomly selected and assigned to negative control group (NG), GHRP-2 injection group (GG) and cysteamine feeding group (CG), with 5 yaks per group. Another five 1-year-old Qinghai high plateau yaks with normal growth performance (average body weight 75.3 ± 2.43 kg) were selected as positive control group (PG). The average daily gain (ADG) of the GG and CG were significantly higher than those in the PG and NG (*P* < 0.05). Both GHRP-2 and CS administration significantly enhanced the myofiber diameter and area of skeletal muscle (*P*<0.05). GHRP-2 significantly enhanced the serum GH and IGF-1 levels (*P* < 0.05), and up-regulated *GHR*, *IGF-1* and *IGF-1R* mRNA expression in the liver and skeletal muscle (*P* < 0.05), enhanced the mRNA expression of *PI*_*3*_*K*, *AKt* and *mTOR* in the skeletal muscle (*P*<0.05). CS significantly reduced the serum SS levels and the hypothalamus *SS* mRNA expression (*P* < 0.05), and enhanced *GHR* and *IGF-1* mRNA expression in the liver (*P* < 0.05), decreased the mRNA expression of *muscle atrophy F-box* (*Atrogin-1*) and *muscle ring finger 1* (*MuRF1*) mRNA (*P* < 0.05). Conclusions: Growth retardation in yaks was primarily due to somatotropic axis hormones secretion deficiency. Both GHRP-2 and CS administration can accelerate growth performance and GH, IGF-1 secretion in yaks with growth retardation. GHRP-2 enhanced muscle protein deposition mainly by up-regulated the protein synthesis pathways, whereas CS worked mainly by down-regulated the ubiquitin-proteasome pathway.

## Introduction

Bovid yaks (*Bos grunniens*) are primarily distributed in the Tibetan plateau. More than 20 million yaks (92 percent of the world population) are found in China. Yaks play an important role in the daily life of local herdsman and represent an important source of meat, milk, wool and fuel. Yak meat is increasingly popular and provides a significant contribution to the local economy, Thus, studies are increasingly focusing on yaks growth performance. However, growth retardation is widespread in yaks due to cold temperatures, food deprivation, malnutrition, various diseases, severe plateau environmental conditions and primitive grazing methods. The phenomenon is similar to that observed in stunted children living in severely poor regions. Malnutrition and disease can cause permanently stunted growth in both children and calves[1–3]. Yaks with growth retardation are associated with extremely low feed conversion efficiency, great morbidity and mortality, which lead to increased yak breeding costs.

Animal somatic growth is primarily controlled by somatotropic axis hormones, which include growth hormone releasing hormone (GHRH), somatostatin (SS), growth hormone (GH), insulin-like growth factors (IGFs) and their carrier proteins and receptors [4]. The significant relationship between GH/IGF-1 and the growth rates of pigs, bovines and humans has been widely reported [5]. Growth retardation is associated with disorders in the secretion of these hormones in animals and humans [6]. However, the relationship between body weight and serum hormones has not been studied on yaks with various growth phenotypes. The amounts of somatotropic axis hormones in stunted growth yaks remain uninvestigated.

Growth hormone releasing peptide-2 (GHRP-2), a member of growth hormone secretagogue, is an analogue of Met-enkephalin in vivo. GHRP-2 can availably stimulate the release of GH and IGF-1, and increase body weight in various species of animal after subcutaneous, intravenous or oral administration [7, 8] and also can improve plasma GH levels and enhance the growth rate of short-stature animals [9] and children [10, 11]. Cysteamine (CS), a metabolite in animals, was used to stimulate the growth rates of fish, pigs and ruminants [12, 13] as a feed additive possibly by exhausting the SS level in the serum and tissue. The potential use of GHRP-2 and CS to stimulate GH secretion and improve growth rate in yaks with growth retardation had not been reported, and the different action methods of the two substances have not been elucidated.

The GH/IGF system plays an important role in the development of skeletal muscle [14]. Skeletal muscle makes up 40% of the animal body weight [15]. The mass of skeletal muscle determine the production performance and economic benefit of livestock. The growth rate of skeletal muscle is significantly associated with daily gain, feed conversion efficiency and the meat percentage of the carcass. Skeletal muscle development primarily depends on increasing the number and hypertrophy of myofibers. The amount of muscle fiber was observed to be constant prior to birth [16]. Therefore, postnatal myofiber development primarily depends on myofiber hypertrophy by increasing protein deposition. The IGF-1 signal pathway, which primarily functions through the PI_3_K / Akt / mTOR pathways, was reported to have the ability to increase myofiber area and muscle hypertrophy by stimulating protein synthesis [17]. Muscle atrophy is primarily due to protein degradation, which is activated by muscle atrophy F-box (Atrogin-1) and muscle ring finger 1 (MuRF1). Atrogin-1 and MuRF1 are muscle-specific E3 ubiquitin ligases of the ubiquitin (Ub)-proteasome pathway [18]. Previous studies reported that IGF-1 had the ability to suppress Atrogin-1 and MuRF1 in atrophying muscle [19, 20]. GHRP-2 can reduce the expression of Atrogin-1 and MuRF1 mRNA in pathology scenarios and stimulate muscle repair [21]. However, the potential of GHRP-2 and CS to promote skeletal muscle development by stimulating GH secretion have not been reported. The objective of this study was to investigate the relationship between body weight and serum hormones levels of yaks and the effects of GHRP-2 and CS administration on growth performance, as well as somatotropic axis hormones and muscle protein deposition in yaks with growth retardation.

## Materials and Methods

### Ethics Statement

All study that involving animals was conducted according to the Regulations for the Administration of Affairs Concerning Experimental Animals (Ministry of Science and Technology, China, revised in June 2004) and approved by the Institutional Animal Care and Use Committee in Sichuan Agricultural University, Sichuan, P. R. China. The animals were humanely sacrificed as necessary to ameliorate suffering.

### Study site

The experiment was carried in Animal Husbandry and Veterinary Institute of Haibei State in Qinghai Province of China during April to August in 2014. The experiment farm is located at 3200 m above mean sea level on 100°54’ East longitude and 36°57’ North latitude. The annual rainfall is about 500 mm and average annual temperature is -0.7°C with a high temperature of 27°C and a low temperature of -39°C. The annual total sunshine is 2440~3140 h and total solar radiation is 131~177 Kcal/cm^2^.

#### Experiment 1

Thirty-six one-year-old Qinghai plateau yaks with varying body weights (38–83.2 kg) were randomly chosen and marked with ear tags. The entire yaks colony was dewormed and vaccinated prior to the trial. The yaks were housed in barns 24 h per day without individual hutches. All yaks were fed the same concentration supplement and oaten hay (concentrate to forage ratio = 35:65) *ad libitum* twice per day at 0900 h and 1700 h. Water was provided at all times.

After a one-week transitional period, body weight and jugular blood samples (10 mL) of the 36 yaks were collected prior to morning feeding. Each blood sample was centrifuged at 3,500 rpm, and serum was collected and stored at -20°C to assay the serum GHRH, GH and IGF-1 levels.

#### Experiment 2

**Animals and experimental design:** Fifteen one-year-old Qinghai plateau yaks with growth retardation (average body weight of 54.8±1.86 kg, 1.5-fold standard deviations less than the average weight of yak colony with same age and breed [22] were randomly divided into 3 groups: a negative control group (NG), a GHRP-2 injection group (GG) and a CS feeding group (CG). Five yaks were put into each group according to weight to maintain an overall even weight distribution between the groups. Another five 1-year-old Qinghai high plateau yaks with normal growth performance (average body weight 75.3 ± 2.43 kg) were selected as a positive control group (PG). The NG was fed basal diet including concentrate and oaten hay everyday. The GG underwent jugular vein injection of 10 ml (40 μg/ml) 12.5 μg/kg BW GHRP-2 solution 2 hours after morning feeding on days 1–6, 11–16 and 21–26 [23], the other administrations were the same as those for the NG. The CG underwent feeding of 5 g/d of cysteamine for each yak for days 1–28, the other administrations were the same as those for the NG. The CS was weighed and premixed in the concentrate everyday. The administration of PG was the same as those for the NG. The yaks were dewormed, vaccinated and marked with ear tags prior to the trial. All trial animals were housed in four pens according to treatment. After a 14-days of adaptation period, an 84-days formal feeding trial was performed. The yaks were fed *ad libitum* on a basal diet at 0900 h and 1700 h each day. Water was provided at all times.

**Basal diet:** The basal diet was designed according to Chinese Beef cattle Raising Standard 2004 (NY/T 815–2004). The concentrate contained 53% corn, 16% soybean meal, 17% wheat bran, 8% rapeseed dregs, 0.8% rapeseed oil, 0.8% salt, 1% baking soda and 3.4% minerals and vitamins. Roughage was oaten hay. The concentrate to roughage ratio of basal diet was 35: 65. Nutrition levels of the basal diet were net energy for gain (NEg) 1.44 Mcal/kg, ether extract (EE) 2.41%, crude protein (CP) 10.70%, acid detergent fiber (ADF) 17.93%, neutral detergent fiber (NDF) 34.92%, calcium (Ca) 0.50% and phosphorus (P) 0.34%.

**Chemicals:** Growth hormone-releasing peptide-2 (GHRP-2) was purchased from Chinapeptides Co., Ltd and made by chemical synthesis way. The chemical structural formula of GHRP-2 is DAla-D-2-Nal-Ala-Trp-DPhe-Lys-NH_2_, purity 95%. GHRP-2 powder was dissolved in normal saline to the concentration of 40 μg/ml and then store at 4°C. CS was brought from Walcom Co., Ltd. The CS purity is 30% which is processing with microcapsule technology.

**Samples collection:** Body weight was measured prior to morning feeding on days 0, 28, 56 and 84. Blood samples of 20 mL were collected from the jugular vein prior to morning feeding on days 0, 28, 56 and 84. After stewing for 30 min, the serum was extracted from blood samples after centrifuging at 3,500 rpm, The serum was then stored at -20°C until analysis.

After an 84-day feeding trial, 4 yaks in each group that were close to the group average weight were slaughtered via captive bolt stunning and exsanguination following the conventional procedures of the Animal Husbandry and Veterinary Institute of Haibei State in Qinghai Province. Immediately after slaughter, the skulls of the yaks were opened with saws, and the whole cerebrums were removed. The hypothalamus and pituitary were collected from the bottom of the cerebrum and packed into 1.5 mL tubes, respectively, after washing with normal saline, and then stored at -80°C. A liver sample was obtained from the same location in each yak’s liver and store at -80°C. A sample of Longissimus dorsi (LD) muscle at the 13th rib and semitendinosus (ST) were removed from the left side of each carcass and packed into 1.5 mL tubes, respectively, and stored at -80°C for gene analysis. Additionally, the same part of the LD and ST muscles was sliced (2*2*2 cm) and immediately stored in 4% paraformaldehyde solution for myofiber morphology analysis.

**Average daily gain (ADG):** The body weight on days 0, 28, 56 and 84 were recorded and the average daily gain (ADG) were calculated following the formula:

$$\mathrm{ADG}=\frac{\left(\mathrm{Final weight}-\mathrm{Initial weight}\right)}{\mathrm{days}}$$

**Serum hormones levels:** The serum samples were measured by the College of Animal Science and Technology, China Agricultural University. Enzyme-linked immunosorbent assay kits (Nanjing Jiancheng Technology Co., Ltd., Nanjing, China) were used to analyze the serum somatotropic axis hormones levels, including GHRH, SS, GH and IGF-1, according to the manufacturer`s instructions. The intra- and inter- assay variations were 15% and 9%, respectively. The absorbance was measured at 450 nm with a microplate reader (MULTISKAN SPECTRUM, Thermo Scientific, USA) within 15 min. PBST was used as the blank control. Each indicator of samples was performed in triplicate with matrixing through the standard curve.

**Muscle fiber characteristics:** The skeletal muscle samples were stored in 4% paraformaldehyde solution and embedded in paraplast after washing and dehydration. Then, the blocks were cut into 5-μm-thick slides using a rotary microtome (RM2235, Leica, Germany). The sections were stained using Hematoxylin and Eosin (H&E) [24]. Each sample for histological analysis was cut into 3 sections. Ten images in random fields of each section were obtained using a Nikon microscope (Eclipse E400, Tokyo, Japan). The myofiber mean diameter and cross-sectional area were calculated from more than 30 myofibers of each image using Image-Pro Plus 6.0 software.

**RNA extraction:** Total RNA in tissues of the hypothalamus, pituitary, liver, LD and ST were extracted using a kit (Takara, Japan) in accordance with the instructions of the manufacturer. RNA purity was determined as the ratio of absorbance at 260/280 nm using a Nano-100 micro-spectrophotometer (NanoDrop Technologies, Wilmington, USA). RNA quality was assessed with 1.5% agarose gel. cDNA was produced from 0.5 μg of RNA using a cDNA Synthesis Kit (Takara, Japan) in accordance with the instructions of the manufacturer. Finally, 40 μL of cDNA was obtained and stored at -20°C for Real-time PCR.

**Real-time PCR:** RT-PCR was carried out using a SYBR Green kit (Takara, Japan) according to the manufacturer`s instructions. The volume of working solution was 12.5 μL including 6 μL of premixed solution, 0.5 μL of forward and reverse primers, 1 μL of cDNA and 4.5 μL of sterilized distilled water. The primers are shown in Table 1. After 3 min at 95°C of activation, the thermal cycler ran for 39 cycles in three steps: denaturing for 10 s at 95°C, annealing for 30 s at the temperature of each primer and extension for 30 s at 72°C. After PCR, a melting curve analysis (10 s at 95°C, 65°C for 1 min followed by increases of 0.5°C /5 s until 95°C) was performed. Negative controls were put in without additional cDNA samples for each PCR. The results were calculated using the 2^-ΔΔCT^ [25] method and presented as the fold-change compared with the NG.

**Table 1:** Information for primers used in the experiment.

Gene or mRNA	Primer sequence (5’-3’)	GenBank accession number	Tm (°C)	Amplicon length (bp)
β-actin		DQ 838049	62.5	127
F	GATCTGGCACCACACCTTCTAC			
r	GATCTGGGTCATCTTCTCACG			
GHRH		NM 178325	62.5	130
f	CTGTCTGCCCGCAAGCTACT			
r	GTGCCATCTGCTGTTGGTCTGT			
SS		NM 31217.1	62.5	119
f	TGATGCCCTGGAGCCTGAA			
r	AGCCAGCTTTGCGTTCTCG			
GH		EF 154193.1	61.5	120
F	TGTTTGCCAACGCTGTGCT			
R	CCTGGGTGTTCTGGATGGAGTA			
GHR		NM 176608	62.5	179
F	GTTTGACAGAGATTCATGCCGAC			
R	CAGTCTCAACGAGTACATCGGAAC			
IGF-1		BC 126802.1	55.9	121
F	ATCAGCAGTCTTCCAACCCAAT			
R	TGAAGGCGAGCAAGCACAG			
IGF-1R		NM 001244612	58.4	163
F	CGGCTCAACCCAGGGAACTA			
R	CCACTATCAACAGAACCGCAATG			
PI_3_K		XM 614711	63.3	87
F	GCCAAGCATTGTTGAAGGGT			
R	GCACCAGCCGATCTACAAAAG			
AKt		NM 173986.2	62.5	100
F	GCCAAGGAGATCATGCAGCA			
R	CAGATGTGACCTGAGGCTTGAA			
mTOR		XM 005901305.1		138
f	CATCATTCGCATTCAGTCCATC			
r	TCCTGCCGCAAGTCCTCAT			
Myostain		EU 926670.1	58.4	100
f	TCTCGATGCTGTCGTTACCCTC			
r	CTCCAGAGCAGTAATTGGCCTT			
Atrogin		NM 001046155.1	62.5	150
F	CTGGTCCAAAGAGTCGGCAAGT			
R	AGGCAGGTCTGTGAAGGTGAGG			
MuRF1		NM 001046295.1	62.5	121
F	ATGCTGGTGGCAGGGAATG			
R	GCGTGTCAAACTTCTGGCTCA			

All primers were designed according to the CDS of the mRNA sequence in NCBI.

**Statistical analyses:** All data were analyzed using SPSS v.19.0. The Spearman Correlation of Coefficients was analyzed between body weight and serum hormone levels. The other data were analyzed by one-way ANOVA followed Duncan’s post-hoc testing. *P*-values between groups less than 0.05 were regarded as statistical significance.

## Results

### Experiment 1

#### Relationship between body weight and somatotropic axis hormones

Firstly, 36 yaks were equally divided into groups I, II and III with body weights ranging from low to high. Each group included 12 yaks. The average body weights of groups I, II and III were significantly different (47.5, 55.8 and 73.8 kg, respectively, *P* < 0.05). The group average serum GHRH, GH and IGF-1 levels increased with the average body weight. The serum GHRH, GH and IGF-1 levels in group II were 31.81%, 26.14% and 28.86% significantly higher than those in group I, respectively (*P* < 0.05). The hormone levels in groups II and III were not significantly different (*P* > 0.05) (Table 2).

**Table 2:** Comparison of body weight and somatotropic axis hormones in yaks from different groups.

	Groups				
Items	Ⅰ	Ⅱ	Ⅲ	SEM	*P*-Value
Number	12	12	12	_	_
BW (kg)	47.5^c^	55.8^b^	73.8^a^	2.504	0.000
GHRH (mg/ml)	0.88^b^	1.16^a^	1.13^a^	0.285	0.035
GH (ng/ml)	74.10^b^	93.47^a^	95.30^a^	3.658	0.026
IGF-1 (ng/ml)	84.21^b^	108.51^a^	113.77^a^	5.057	0.033

I, II, III: 36 yaks were equally divided into groups I, II and III with body weights ranging from low to high; Number: the number of yaks in each group; BW: body weight; SEM: standard error of the mean. Data with different small letter superscripts within the same row are significantly different (*P*< 0.05).

The relationship between the body weight and the serum GHRH, GH, IGF-1 levels of 36 yaks was not significantly correlated (*P* > 0.05). The relationship between each somatotropic axis hormone was significantly correlated (*P* < 0.05) (Table 3).

**Table 3:** Relationship between body weight and somatotropic axis hormone level (n = 36).

	Correlation coefficient (*P*-Value)			
Items	Body weight	GHRH	GH	IGF-1
Body weight	1	0.18 (0.336)	0.24 (0.209)	0.19 (0.314)
GHRH		1	0.84 (0.000)	0.84 (0.000)
GH			1	0.80 (0.000)
IGF-1				1

The sample size in Table 3 was 36 yaks; *P*-values of less than 0.05 were regarded as significant correlations.

**Fig 1:**
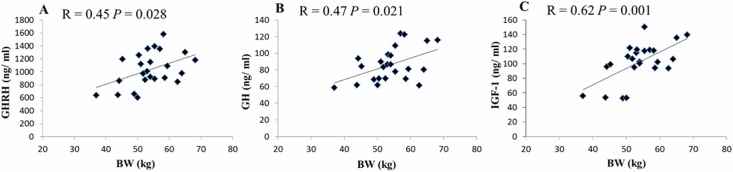
Relationship between body weight and somatotropic axis hormone level of yaks in groups I and II (n = 24) A is the relationship between the body weight and serum GHRH levels; B is the relationship between the body weight and serum GH levels; C is the relationship between the body weight and serum IGF-1 levels; *P*-values of less than 0.05 indicated significant correlation.

The relationships between body weight and serum GHRH, GH, IGF-1 levels of the yaks in groups I and II (n = 24) were significantly correlated (*P* < 0.05). The relationship between body weight and IGF-1 levels had the highest correlation (R = 0.62, *P* = 0.001) (Fig 1).

### Experiment 2

#### Effects of GHRP-2 and CS on growth performance in yaks with growth retardation

GHRP-2 injection and CS feeding improved growth performance in yaks with growth retardation. (Table 4) The initial weight of the yaks in NG, GG and CG were not significantly different and were significantly less than those in the PG (*P* < 0.05). After 84 days of feeding, the final weights of the yaks in the GG and CG were significantly more than that in the NG (*P* < 0.05) and were close to the weight of normal yaks (*P* > 0.05). The yaks in the GG and CG had a significantly higher ADG than the NG and PG throughout the study (*P* < 0.05). The ADG of the GG were the highest (*P* < 0.05) during days 0–28 but fell during days 29–84 after stopping GHRP-2 injection. The ADG of the CG were lower than those in the GG during days 0–28 but had the highest ADG (*P* < 0.05) during days 29–84 with a continuously increasing tendency.

**Table 4:** Effects of GHRP-2 and CS on growth performance in yaks with growth retardation.

	Treatments					
Items	NG	GG	CG	PG	SEM	*P*-Value
0d BW(kg)	53.8^b^	55.6^b^	55.4^b^	75.4^a^	2.61	0.001
28d BW(kg)	60.6^b^	68.6^b^	65.8^b^	84.0^a^	2.71	0.002
56d BW(kg)	67.8^c^	79.7^b^	77.9^bc^	93.2^a^	2.89	0.004
84d BW(kg)	75.2^b^	91.1^a^	90.5^a^	102.9^a^	3.11	0.002
0–84 d ADG(kg/d)	0.26^c^	0.42^a^	0.42^a^	0.33^b^	0.017	0.027
0–28 d ADG(kg/d)	0.24^c^	0.47^a^	0.37^ab^	0.31^b^	0.017	0.002
29–56 d ADG(kg/d)	0.26^c^	0.400^ab^	0.43^a^	0.33^b^	0.020	0.027
57–84 d ADG(kg/d)	0.26^c^	0.41^ab^	0.45^a^	0.35^b^	0.024	0.000

NG: negative control group (yaks with growth retardation being fed basic rations); GG: GHRP-2 injection group (yaks with growth retardation being fed basic rations and GHRP-2 injection on days 1–6, 11–16, and 21–26); CG: CS feeding group (yaks with growth retardation being fed basic rations and supplementary fed cysteamine on days 1–28); PG: positive control group (yaks with normal growth being fed basic rations); SEM: standard error of the mean. Data with different small letter superscripts within the same row are significantly different (*P*<0.05). BW: body weight; ADG: average daily gain.

#### Effects of GHRP-2 and CS on somatotropic axis hormone in yaks with growth retardation

**Fig 2:**
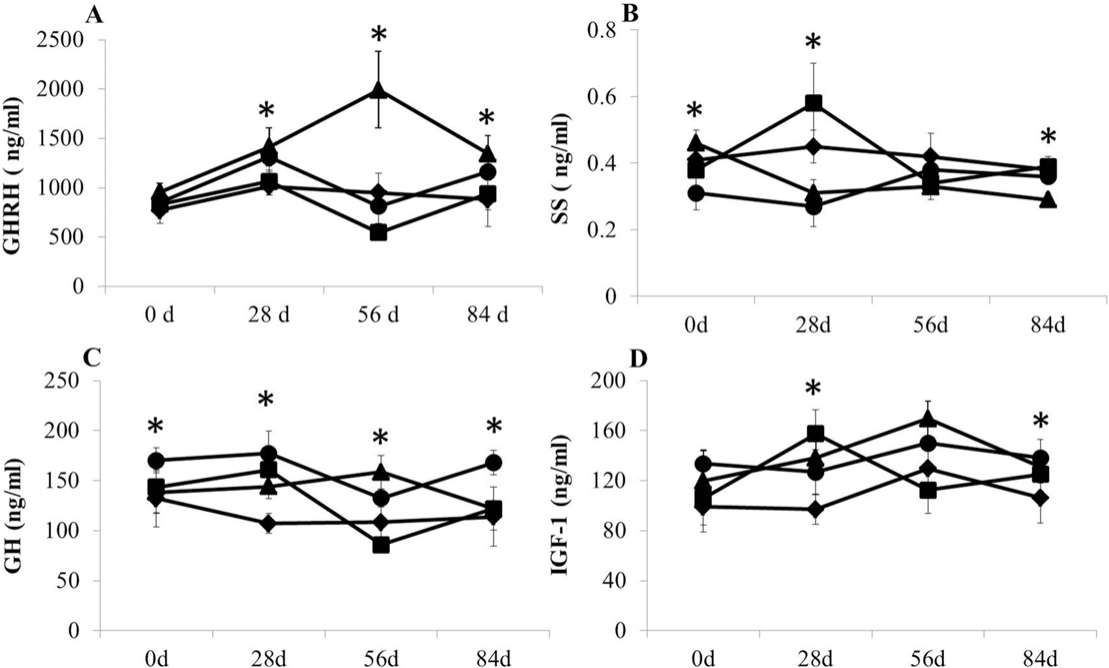
Effects of GHRP-2 and CS on serum hormone levels in yaks with growth retardation A is the serum GHRH levels; B is the serum SS levels; C is the serum GH levels; D is the serum IGF-1 levels; Rhombus: negative control group (yaks with growth retardation being fed basic rations); Square: GHRP-2 injection group (yaks with growth retardation being fed basic rations and GHRP-2 injection on days 1–6, 11–16, and 21–26); Triangle: CS feeding group (yaks with growth retardation being fed basic rations and supplementary fed cysteamine on days 1–28); Roundness: positive control group (yaks with normal growth feeding basic ration); “*” indicates significant differences (*P* < 0.05).

CS supplementary feeding significantly enhanced the serum GHRH levels (*P* < 0.05) and significantly reduced the serum SS levels (*P* < 0.05) in yaks with growth retardation as measured on days 28, 56 and 84 (Fig 2). GHRP-2 injection did not significantly influence serum GHRH levels (*P* > 0.05). The serum GH and IGF-1 levels in the yaks in the GG were significantly enhanced on day 28 after three rounds of GHRP-2 injection (*P* < 0.05). GH and IGF-1 levels of the CG were somewhat lower than those in the GG and markedly higher than those in the NG on day 28 (*P* < 0.05). The GH and IGF-1 levels decreased sharply on day 56 (*P* < 0.05) and increased on day 84 in the GG. The CG had the highest GH and IGF-1 levels in the four groups on day 56 (*P* < 0.05), this had declined by day 84.

Gene expressions are shown in Table 5. CS supplementary feeding significantly reduced the *SS* mRNA expression in the hypothalamus in yaks with growth retardation (*P* < 0.05). GHRP-2 injection and CS supplementary feeding significantly enhanced the expression of *GHR* and *IGF-1* mRNA in the liver (*P* < 0.05). The impact of GHRP-2 was stronger than that of CS. Additionally, GHRP-2 injection significantly enhanced the expression of *GHR*, *IGF-1* and *IGF-1R* mRNA in skeletal muscle (*P* < 0.05), whereas CS had no significant effects (*P* < 0.05).

**Table 5:** The effects of GHRP-2 and CS on somatotropic axis hormone gene expression in yaks with growth retardation.

		Treatments					
Genes	Tissues	NG	GG	CG	PG	SEM	*P*-Value
GHRH	Hypothalamus	1.00	1.28	1.12	1.33	0.121	0.221
SS	Hypothalamus	1.00^b^	1.87^a^	0.84^b^	1.07^b^	0.063	0.003
GH	Pituitary	1.00	1.24	0.98	1.16	0.092	0.103
GHR	Liver	1.00^c^	1.85^a^	1.56^b^	1.21^bc^	0.108	0.002
	LD	1.00^b^	2.15^a^	1.29^b^	0.89^b^	0.135	0.015
	ST	1.00^ab^	1.33^a^	0.73^b^	1.12^ab^	0.076	0.032
IGF-1	Liver	1.00^c^	3.08^a^	2.40^b^	1.80^bc^	0.253	0.000
	LD	1.00^ab^	1.60^a^	0.86^b^	1.12^ab^	0.056	0.007
	ST	1.00^bc^	1.93^a^	0.89^c^	1.25^b^	0.051	0.003
IGF-1R	Liver	1.00^b^	1.63^a^	1.21^ab^	1.55^a^	0.026	0.008
	LD	1.00^c^	2.92^a^	1.17^c^	1.76^b^	0.028	0.002
	ST	1.00^c^	3.80^a^	1.07^c^	1.87^b^	0.107	0.002

NG: negative control group (yaks with growth retardation being fed basic rations); GG: GHRP-2 injection group (yaks with growth retardation being fed basic rations and GHRP-2 injection on days 1–6, 11–16, and 21–26); CG: CS feeding group (yaks with growth retardation being fed basic rations and supplementary fed cysteamine on days 1–28); PG: positive control group (yaks with normal growth being fed basic rations); LD: Longissimus dorsi; ST: semitendinosus; SEM: standard error of the mean. Data with different small letter superscripts within the same row are significant different (*P*<0.05).

#### Effects of GHRP-2 and CS on skeletal muscle protein deposition in yaks with growth retardation

**Fig 3:**
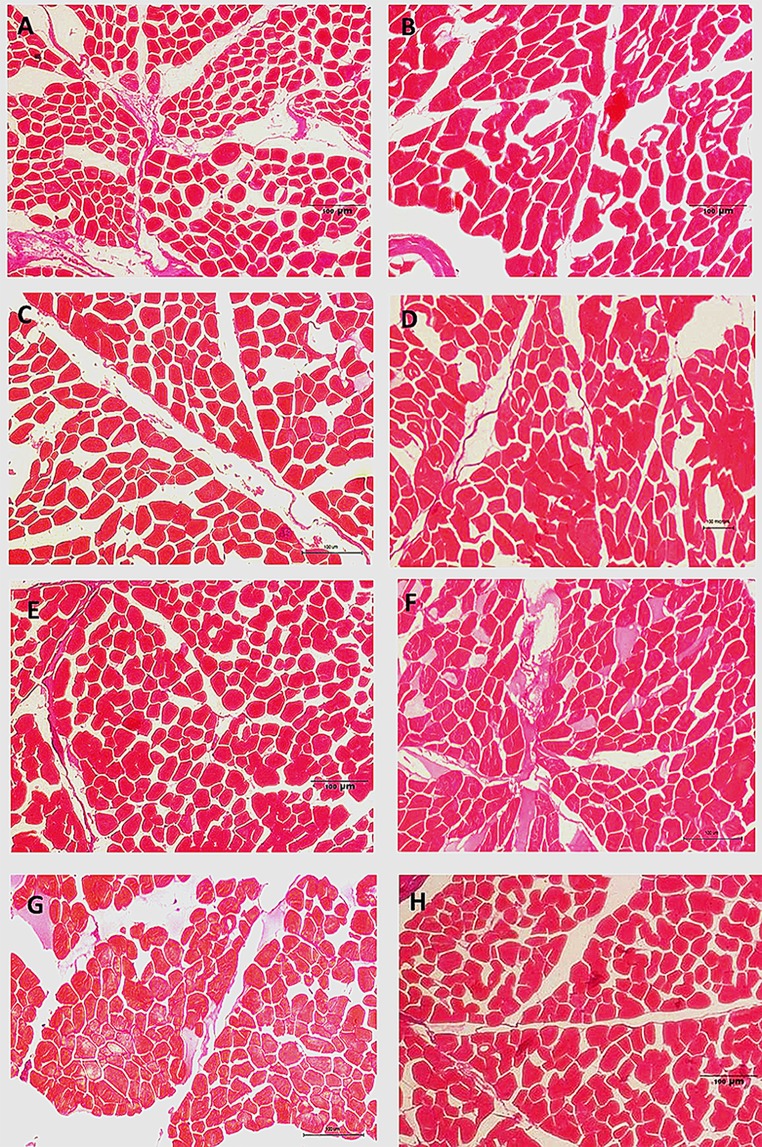
Effects of GHRP-2 and CS on the myofiber parameters in yaks with growth retardation A, B, C and D represent Longissimus dorsi samples in the NG, GG, CG and PG, respectively; E, F, G and H represent the semitendinosus samples in the NG, GG, CG and PG, respectively; Each picture was obtained via microscope with 200 x magnification.

The myofiber of yaks in the GG and CG were significantly thicker compared with those in NG (*P* < 0.05) but were similar to that in normal growth yaks. The myofiber diameter and area of LD in the CG had a tendency to be higher than that in the GG (*P* > 0.05). In ST, the myofiber diameter and area of CG were lower than those in the GG (without significance, *P* > 0.05). (Table 6 and Fig 3).

**Table 6:** The effects of GHRP-2 and CS on the myofiber parameters in yaks with growth retardation.

		Treatments					
Items	Tissue	NG	GG	CG	PG	SEM	*P*-Value
Muscle fiber diameter(um)	LD	21.27^c^	28.08^b^	30.87^ab^	32.05^a^	1.397	0.002
	ST	24.76^b^	31.59^a^	30.28^a^	31.58^a^	1.232	0.031
Cross-sectional area(um^2^)	LD	398.42^b^	733.75^a^	822.05^a^	921.73^a^	33.420	0.005
	ST	530.91^b^	888.97^a^	837.56^a^	871.85^a^	40.617	0.048

NG: negative control group (yaks with growth retardation being fed basic rations); GG: GHRP-2 injection group (yaks with growth retardation being fed basic rations and GHRP-2 injection on days 1–6, 11–16, and 21–26); CG: CS feeding group (yaks with growth retardation being fed basic rations and supplementary fed cysteamine on days 1–28); PG: positive control group (yaks with normal growth being fed basic rations); LD: Longissimus dorsi; ST: semitendinosus; SEM: standard error of the mean. Data with different small letter superscripts within the same row are significant different (*P*<0.05).

**Fig 4:**
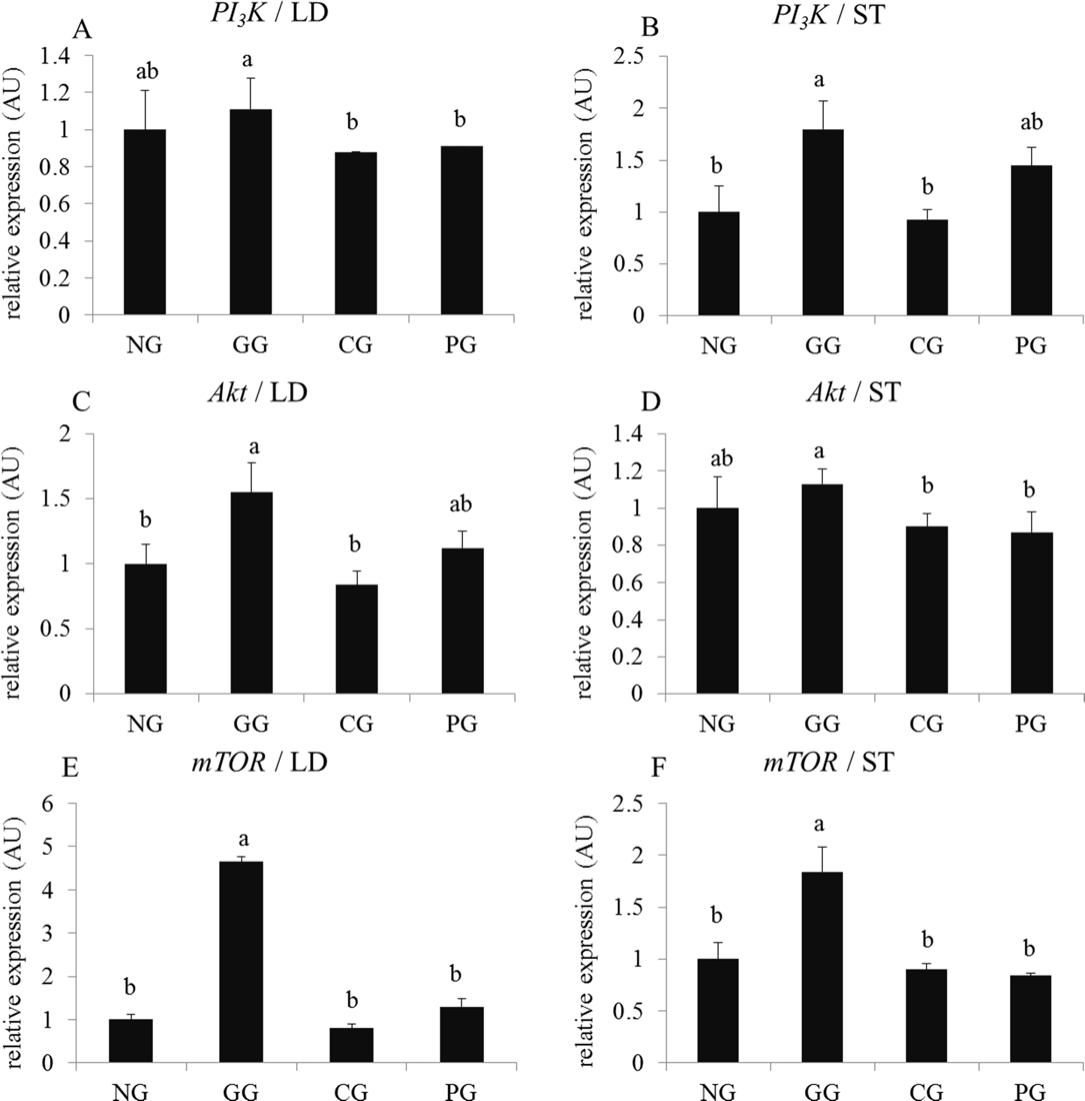
Effects of GHRP-2 and CS on the genes relative expression of the *PI*_*3*_*K* / *Akt* / *mTOR* pathways in the LD and ST in yaks with growth retardation A, C and E show the *PI*_*3*_*K*, *Akt* and *mTOR* mRNA relative expression in Longissimus dorsi (LD), respectively; B, D and F show the *PI*_*3*_*K*, *AKt* and *mTOR* mRNA relative expression in semitendinosus (ST), respectively; NG: negative control group (yaks with growth retardation being fed basic rations); GG: GHRP-2 injection group (yaks with growth retardation being fed basic rations and GHRP-2 injection on days 1–6, 11–16, and 21–26); CG: CS feeding group (yaks with growth retardation being fed basic rations and supplementary fed cysteamine on days 1–28); PG: positive control group (yaks with normal growth being fed basic rations); The bars with different small letter superscripts within the same graph are significant different (*P* < 0.05).

As shown in Fig 4, GHRP-2 administration dramatically enhanced the mRNA expression of the *PI*_*3*_*K* / *Akt* / *mTOR* pathway (*P* < 0.05). *mTOR* mRNA expression in the LD of GG increased approximately 5-fold compared with the NG and CG (*P* < 0.05). However, CS administration did not significantly affect the mRNA expression of these key factors affecting the muscle protein synthesis pathway compared with NG (*P* > 0.05).

**Fig 5:**
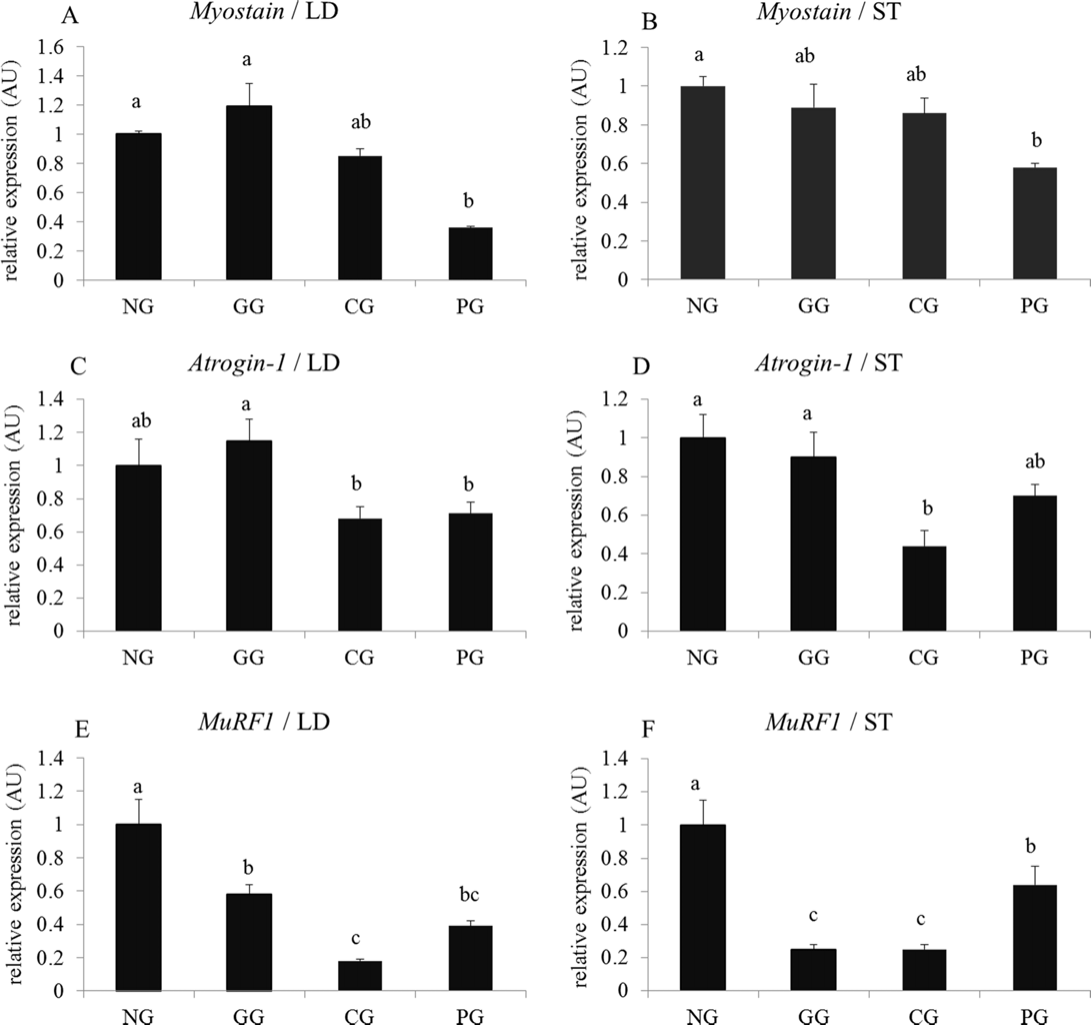
Effects of GHRP-2 and CS on genes relative expression of the protein degradation pathway in the LD and ST in yaks with growth retardation A, C and E are the relative expression of *Myostain*, *Atrogin-1* and *MuRF1* mRNA in the Longissimus dorsi (LD), respectively; B, D and F are the relative expression of *Myostain*, *Atrogin-1* and *MuRF1* mRNA in the semitendinosus (ST), respectively; NG: negative control group (yaks with growth retardation being fed basic rations); GG: GHRP-2 injection group (yaks with growth retardation being fed basic rations and GHRP-2 injection on days 1–6, 11–16, and 21–26); CG: CS feeding group (yaks with growth retardation being fed basic rations and supplementary fed cysteamine on days 1–28); PG: positive control group (yaks with normal growth being fed basic rations); The bars with different small letter superscripts within the same graph are significant different (*P* < 0.05).

As shown in Fig 5, the *Myostain* and *MuRF1* mRNA expression in the LD and ST of the PG were significantly lower than those in the NG (*P* < 0.05). GHRP-2 and CS administration did not markedly influence the expression of *Myostain* mRNA (*P* > 0.05). The *Atrogin-1* mRNA expression in the LD and ST of the CG were the lowest (*P* < 0.05). GHRP-2 administration did not significantly influence *Atrogin-1* mRNA expression in yaks with growth retardation (*P* > 0.05). GHRP-2 and CS administration significantly reduced the expression of *MuRF-1* mRNA (*P* < 0.05), CS had a stronger inhibitory effect than GHRP-2.

## Discussion

### Relationship between body weight and somatotropic axis hormones

Somatotropic axis components play important roles in animal growth. GH / IGF-1 system hormone secretion deficiencies and disorders are often associated with growth retardation. IGF-1 deletion mutant mice had more severe growth restriction than GHR deletion mice. GHR / IGF-1 double-mutant mice had the most severe restrictions and were only 17% of their normal body weight [6]. In the experiment, 36 yaks were equally divided into three groups according to body weight ranging from low to high. The serum GHRH, GH and IGF-1 levels significantly increased as the average group body weight increased. The somatotropic axis hormone levels were associated with the growth status of yaks. Yaks with lighter body weight had lower somatotropic axis levels. Graham et al (2010) reported that 40% lower growth rate cattle had significantly lower IGF-1 levels than 40% higher growth rate cattle in a cattle colony [26]. Brickell et al (2009) divided cattle into three groups according to growth rates from low to high and found that the group average body weight and IGF-1 levels significantly increased according to increasing growth rate [27]. However, the hormone level differences between groups I and II were larger than the differences between groups II and III. This suggested that somatotropic axis hormone deficiencies were more severe in yaks with lighter body weight.

There was no significant correlation between somatotropic axis hormones and body weight in this colony of 36 yaks. However, a significant correlation between GHRH, GH, IGF-1 and body weight were found when analysing the data of 24 yaks in groups I and II and the results were accord with the larger hormones difference between groups I and II. IGF-1 levels were the most strongly associated with body weight among the three hormones. IGF-1 is located at the bottom of the somatotropic axis and plays an influential role in cell growth and differentiation [28]. The average serum IGF-1 concentration was positively associated with growth rate, gain:feed ratio and growth performance in pigs [5] and ruminants [29, 30]. Brickell et al (2009) reported that serum IGF-1 was positively associated with heifer growth in the rearing period [27]. That is most likely to the promote growth function of IGF-1 for developing animal long bone [31] and skeletal muscle accretion [32]. GH is an upstream hormone in the somatotropic axis and had a milder correlation with animal growth than IGF-1. Sarkar et al (2008) reported that serum GH levels were significantly correlated with growth in a female yak colony (r = 0.73, *P* < 0.05) [33]. The somatotropic axis hormones were more strongly correlated with the growth status of yaks with lower body weight. The yaks with growth retardation were primarily restricted by somatotropic axis hormone deficiency, which did not represent the most restricted growth factor for yaks with a higher body weight.

Yaks are distributed in a harsh, high-altitude environment. These animals suffer from food deprivation and disease during pregnancy and as neonates in the cold season. Previous studies indicated that fetal and neonatal malnutrition reduced serum GH / IGF-1 levels and induced growth retardation [26]. Incorrect management methods and harsh environments reduced serum IGF-1 levels and growth rates [27]. Therefore, yaks with growth retardation showed somatotropic axis hormone secretion deficiency, which was primarily caused by the severe plateau environment and primitive grazing methods.

### Effects of GHRP-2 and CS on growth performance in yaks with growth retardation

GHRP-2 is a peptide of growth hormone secretagogues (GHS) that were synthesized in reference to the structure of met-enkephalin [34]. The peptide acts on an internal receptor (GHS-R), which ghrelin is a natural ligand for, to stimulate GH secretion [35]. Cysteamine is an intermediate metabolite and is used to enhance the serum GH level by exhausting somatostatin. Cysteamine is used as a feed additive to improve livestock growth performance. The results of the administration of GHRP-2 or CS to promote normal animal growth have been reported in several studies. Phung et al (2000) reported that the chronic s.c. injection of GHRP-2 improved ADG and the feed sufficiency of swine by 22.35% and 20.64%, respectively [36]. The chronic administration of GHRP-2 significantly promoted ADG in Holstein calves by 36.4% but did not influence food efficiency [37]. Dietary cysteamine feeding promoted growth performance, improved health conditions and enhanced protein deposition in pigs [38, 39]. However, the growth-promoting effects of GHRP-2 and CS in stunted growth yaks had not previously been reported. In the present experiment, GHRP-2 and CS improved the growth rates of yaks with growth retardation. The final weight of yaks in the GG and CG were similar, and both were significantly heavier than the yaks in the NG. However, the growth curves of yaks in the GG and CG were different. ADG in the GG was higher than that in the CG by 25.7% during days 0–28. The growth rate in the GG subsequently slowed down, whereas the growth rate in the CG steadily increased during days 29–84. These results indicated that CS administration exerted significantly longer subsequent growth-promoting effects than compared with GHRP-2. Joh et al (1996) reported that the ADG of calves was reduced during days 8–14 compared with days 0–7 during continuous GHRP-2 injection [37]. They speculated that the somatic growth response to GHRP-2 administration became desensitized after long-term repeated injection.

### Effects of GHRP-2 and CS on the somatotropic axis hormone in yaks with growth retardation

The GH secretion of pituitary cells is primarily controlled by the GHRH and SS produced in the hypothalamus, of which GHRH is dominant. Serum GH combines with GHR distributed on the surface of the liver or other tissue and induces IGF-1 generation. IGF-1 acts on IGF-1R in target tissues and activates a downstream biochemical response. GHRP-2 is known to have a strong GH-stimulating activity and is useful in GH insufficiency therapy. GHRP-2 acts on both the hypothalamus and pituitary because GHRPs receptors are located in both locations [40, 41]. GHRP-2 administration improved the serum GH level of neonatal rats with growth retardation caused by hypothalamic dysfunction [9, 42] and in children with multiple forms of short stature [43]. In the present study, GHRP-2 administration dramatically promoted serum GH and IGF-1 levels in yaks with growth retardation. Numberous experiments on GHRP-2 administration stimulating endogenous GH secretion in cattle have been reported [44]. Lee et al (2005) reported that after intravenous (i.v.) injection of GHRP-2, serum GH levels were increased in a dose-dependent manner and reached a peak value at 15 min, before dropping to baseline levels at 90 to 120 min [7, 23]. As the GH receptor in the liver, serum IGF-1 is typically increased along with GH increases during GHRP-2 administration.

A structure of disulfide bonds that is important to the role of SS is located in the SS molecule. CS can break down the disulfide bonds and form a compound with SS [45]. The depletion of SS was considered a fundamental mechanism in CS to enhance the serum GH level. CS feeding significantly promoted serum GHRH, GH and IGF-1 concentrations and reduced serum SS levels in yaks with growth retardation. McLeod et al (1995) found that CS administration in the abomasum also enhanced the serum GH level and reduced the SS concentration in serum and abomasum tissue [46]. Previous studies reported that SS can stimulate the synthesis of β-endorphin (β-END) in hypothalamic arcuate nucleus. β-END increases can stimulate GHRH secretion and enhance serum GH levels.

GHRP-2 and CS have a similar function in enhancing serum GH and IGF-1 levels but with different characteristics. In the present study, somatotropic axis hormones of yaks administered GHRP-2 were sharply reduced after stopping injection and were lower than those in the NG yaks at day 56. However, the hormones in the CG had a steady and high level during days 29–84. This phenomenon implied that CS exerted a sustained and significant action that enhanced GH levels and that long-term GHRP injection may induce GH exhaustion in pituitary or GHRP receptor desensitization. The serum GH response to continuous GHRP-2 i.v. and s.c. injection was significantly attenuated in pigs [36]. Nou et al (2003) reported that GHRP-2 injection twice daily for 10 days significantly reduced serum GH concentrations in pigs during days 6 to 10. The acute administration of GHRP-2 also decreased the serum GH peak values after continuous injection every two hours. Conversely, Phung et al (2000) found that the short-term administration GHRP-2 did not induce the desensitization of GH secretion [47]. The degree of desensitization is related to the animal species, the external environment and the method of GHRP-2 administration.

GHRP-2 and CS administration also significantly affected the gene expression of the somatotropic axis. CS significantly reduced the expression of *SS* mRNA in the hypothalamus. CS can regulate SS on the gene expression level and can exhaust the serum SS. Papachristou [48] reported that CS can influence *SS* mRNA expression in the hypothalamus. After 2 h of CS injection, *SS* mRNA expression increased by 60%. After 8 h, *SS* mRNA was reduced by 55% compared with the control group. In the somatotropic axis peripheral tissue, GHRP-2 and CS significantly promoted *GHR* and *IGF-1* mRNA expression in the liver. 80% of serum IGF-1 is produced by the liver. These results suggested that GHRP-2 and CS can promote IGF-1 endocrinology in yaks with growth retardation and that the impact of GHRP-2 was stronger than that for CS. GHRP-2 can promote *GHR*, *IGF-1* and *IGF-1R* mRNA expression in the skeletal muscle. Granado reported that GHRP-2 enhanced *IGF-1* mRNA expression in the liver, skeletal muscle and myocardium of mice [49]. Giyama found that ghrelin also promoted *IGF-1* mRNA expression in the skeletal muscle and improved nutrition metabolism [50]. They found that GHRP-2 induced both autocrine and paracrine IGF-1 in skeletal muscle.

### Effects of GHRP-2 and CS on skeletal muscle protein deposition in yaks with growth retardation

Skeletal muscle mass has an important influence on the growth performance and meat yield of livestock. The myofiber numbers of animals are nearly invariable after birth. Myofiber diameter and cross-sectional area increases are the basis of skeletal muscle growth after animal birth. The postnatal skeletal muscle growth primarily relies on protein accumulation in myofibers. Increasing serum GH and IGF-1 levels can active the IGF-1 receptor located in the skeletal muscle and can induce the activation of downstream factors to up-regulate protein accumulation. Bodine et al reported that the mechanism of IGF-1-induced myofiber hypertrophy is primarily through the activation of the PI_3_K / Akt / mTOR pathway and increases the phosphorylation of 4E binding protein (4EBP-1) and S6 kinase (S6K1) to stimulate muscle protein synthesis [51, 52]. mTOR is an important factor for muscle cell growth that is affected by the GH / IGF-1 system, nutrition and energy balance. The possibility of GHRP-2 and CS administration stimulating skeletal muscle growth has not been previously reported. In this experiment, GHRP-2 administration significantly enhanced the myofiber morphological parameters and up-regulated the expression of *PI3K*, *AKt* and *mTOR* mRNA in LD and ST. GHRP-2 injection was demonstrated to improve skeletal muscle growth in yaks with growth retardation primarily by increasing protein synthesis. However, we found that CS administration can enhance the myofiber diameter and area, and this did not affect the *PI*_*3*_*K* / *Akt* / *mTOR* pathway compared with that in the NG. This implied that CS may have increased skeletal muscle protein accumulation by inhibiting proteolysis.

MuRF1 and Atrogin-1, which are specific E3 ubiquitin ligases in muscle, are responsible for proteolysis and induced skeletal muscle atrophy [53, 54]. These genes link ubiquitin proteins to target proteins and form complexes. The complexes are then broken down by proteasome which is a special structure in the cell. This pathway is the principal mechanism for skeletal muscle protein degradation and mass reduction. In the experiment, the two genes were used as markers to determine the extent of LD and ST proteolysis following GHRP-2 and CS administration. We observed a sharp decrease in *MuRF-1* and *Atrogin-1* mRNA expression in LD and ST with CS administration. GHRP-2 administration significantly down-regulated the mRNA expression of *MuRF-1* in LD and ST but did not substantially affect *Atrogin-1* mRNA. These results demonstrated that GHRP-2 and CS administration had the ability to decrease skeletal muscle proteolysis and that CS more strongly inhibited the ubiquitin-proteosome pathway than GHRP-2. Previous studies reported that GHRP-2 can attenuate pathology-induced *MuRF-1* and *Atrogin-1* mRNA expression and can help maintain patient body weight [49, 55]. However, in our study, GHRP-2 appeared to exert weaker inhibition effects on E3 ubiquitin ligase compared with CS. These results may be due to the chronic injection of GHRP-2 for 18 days. Sheriff (2009) reported that prolonging GHRP-2 administration may bring E3 ubiquitin ligases to baseline levels [49].

Protein accumulation is the result of a dynamic balance between protein synthesis and degradation [56]. Both GHRP-2 and CS administration can significantly increase myofibers diameter and area that demonstrate increased muscle protein accumulation. In this study, GHRP-2 strongly stimulated the protein synthesis pathway and restrained proteolysis in skeletal muscle, whereas CS strongly inhibited muscle protein breakdown without significantly affecting protein synthesis. CS acting only on the proteolysis pathway was also reported in whole-body protein turnover research. CS supplementation was found to enhance the whole-body net protein gain of pigs by significantly reducing the protein breakdown rate without affecting the protein synthesis rate [13].

## Conclusions

This experiment provided the first evidence indicating that somatotropic axis hormone secretion deficiency was the primary reason for growth retardation in yaks. GHRP-2 and CS administration improved serum GH and IGF-1 levels and the tissue gene expression, skeletal muscle protein deposition and growth performance of yaks with growth retardation. However, GHRP-2 and CS exhibited differing mechanisms of action. GHRP-2 enhanced serum GH and IGF-1 levels, induced *GHR*, *IGF-1* and *IGF-1R* mRNA in the liver and skeletal muscle, and stimulated muscle protein deposition primarily by enhancing protein synthesis and inhibiting protein degradation, whereas CS enhanced serum GHRH levels, reduced serum SS levels and hypothalamus *SS* mRNA expression, enhanced *GHR* and *IGF-1* mRNA in the liver, and stimulated muscle protein deposition primarily by inhibiting protein breakdown.
